# LRP16 Integrates into NF-κB Transcriptional Complex and Is Required for Its Functional Activation

**DOI:** 10.1371/journal.pone.0018157

**Published:** 2011-03-31

**Authors:** Zhiqiang Wu, Yazhuo Li, Xiaolei Li, Dongdong Ti, Yali Zhao, Yiling Si, Qian Mei, Po Zhao, Xiaobing Fu, Weidong Han

**Affiliations:** 1 Department of Molecular Biology, Institute of Basic Medicine, Chinese PLA General Hospital, Beijing, China; 2 Department of Pathology, Chinese PLA General Hospital, Beijing, China; St. Georges University of London, United Kingdom

## Abstract

**Background:**

Nuclear factor κB (NF-κB)-mediated pathways have been widely implicated in cell survival, development and tumor progression. Although the molecular events of determining NF-κB translocation from cytoplasm to nucleus have been extensively documented, the regulatory mechanisms of NF-κB activity inside the nucleus are still poorly understood. Being a special member of macro domain proteins, LRP16 was previously identified as a coactivator of both estrogen receptor and androgen receptor, and as an interactor of NF-κB coactivator UXT. Here, we investigated the regulatory role of LRP16 on NF-κB activation.

**Methodology:**

GST pull-down and coimmunoprecipitation (CoIP) assays assessed protein-protein interactions. The functional activity of NF-κB was assessed by luciferase assays, changes in expression of its target genes, and its DNA binding ability. Annexin V staining and flow cytometry analysis were used to evaluate cell apoptosis. Immunohistochemical staining of LRP16 and enzyme-linked immunosorbent assay-based evaluation of active NF-κB were performed on primary human gastric carcinoma samples.

**Results:**

We demonstrate that LRP16 integrates into NF-κB transcriptional complex through associating with its p65 component. RNA interference knockdown of the endogenous LRP16 in cells leads to impaired NF-κB activity and significantly attenuated NF-κB-dependent gene expression. Mechanistic analysis revealed that knockdown of LRP16 did not affect tumor necrosis factor α (TNF-α)-induced nuclear translocation of NF-κB, but blunted the formation or stabilization of functional NF-κB/p300/CREB-binding protein transcription complex in the nucleus. In addition, knockdown of LRP16 also sensitizes cells to apoptosis induced by TNF-α. Finally, a positive link between LRP16 expression intensity in nuclei of tumor cells and NF-κB activity was preliminarily established in human gastric carcinoma specimens.

**Conclusions:**

Our findings not only indicate that LRP16 is a crucial regulator for NF-κB activation inside the nucleus, but also suggest that LRP16 may be an important contributor to the aberrant activation of NF-κB in tumors.

## Introduction

Nuclear factor κB (NF-κB) is a dimeric transcription factor (p65–p50) that is ubiquitously expressed and highly regulated [1]. Normally, it is localized in the cytoplasm as an inactive complex through physical association with its inhibitor IκBα. A myriad of endogenous and exogenous stimuli, such as tumor necrosis factor α TNF-αand interleukin 1β IL-1β are capable of inducing the activation of IκB kinase (IKK) complex, which then leads to the ubiquitin-dependent degradation of IκBα. NF-κB is then free to shuttle into the nucleus and to bind to specific sequences in the promoter or enhancer regions of their target genes [2], [3]. NF-κB has a central role in the control of genes involved in cell survival, immunity, inflammation, and development [3], [4], [5]. Aberrant activation of NF-κB has been implicated in a variety of diseases, such as atherosclerosis and diabetes [6]. The excessive or constitutive activation of NF-κB is also frequently observed in multiple tumors and this status is positively linked to poor prognosis of those involved tumor patients [6], [7]. To date, NF-κB and the signaling pathways involved in its activation are considered the most attractive targets for cancer prevention and therapy [8]. Several IKK inhibiting compounds have been characterized as effective for inhibiting NF-κB activity in cultured cancer cells and animal models, and are likely to be safe for use in humans [9], [10].

Although the molecular events that control the translocation of NF-κB from the cytoplasm to the nucleus are well characterized [3], knowledge concerning the regulation of NF-κB's activity inside the nucleus still remains largely unknown. NF-κB is believed to recruit a couple of cofactors to form a much higher order transcription complex than once expected [2]. Among the well-characterized NF-κB coactivators, p300/CREB-binding protein (CBP) appear to be the basal components of functional NF-κB transcription complex [11], [12]. Some NF-κB coactivators such as poly(ADP-ribose) polymerase-1 (PARP-1) and coactivator-associated arginine methyltransferase (CARM1), in concert with p300/CBP, synergistically coactivate NF-κB-mediated transcription [13], [14].

The macro domain (130-190 amino acids) is a highly conserved protein module found in all organisms [15], so named because it was initially described as the non-histone region of the variant histone macroH2A [16]. In vertebrates, macro domains are frequently found in tandem with modules involved in ADP ribosylation or the polymerization of poly(ADP-ribose) (PAR) as well as ATP-dependent chromatin remodeling [15], [17]. Although structure analysis indicated that macro domain cannot directly bind DNA [18], several macro domain proteins were found to be able to access chromatin through sensing the activation of PARP-1's enzyme activity [19], [20], and a few macro domain proteins (only CoaSt6 and LRP16) were found to selectively bind to chromatin with specific transcription factors and regulate their transcriptional activities [21], [22], [23].

LRP16 can be regarded as a special member of the macro domain superfamily because only a single stand-alone macro module is harbored by it at its C-terminal region [17], [23]. Biochemical characteristics analysis revealed that LRP16 can bind with ADP-ribose metabolites including both mono(ADP-ribose) and PAR by its macro domain module [24]. Similar with other macro domain proteins, LRP16 can be recruited to the DNA damage sites mediated by its biochemical capacity to bind PAR [20]. More importantly, LRP16 has a specific function in that it acts as a coactivator of both estrogen receptor α (ERα) and androgen receptor (AR), as well as being their target gene [22], [23], [25].

UXT, a putative member of an α-class prefoldin protein family, was reported to be able to interact with NF-κB and enhance its transcriptional activity by the mechanism of maintaining the presence of NF-κB inside the nucleus [26]. Independently, we identified the physical interaction of LRP16 and UXT *in vitro* and *in vivo* by a series of experiments [23]. On the basis of these findings, we proposed that LRP16 may also regulate NF-κB activity. To test this hypothesis, we used TNF-α-sensitive 293T and HeLa cells to investigate the LRP16 regulation of TNF-α-induced NF-κB activity. In this study, we provide evidence to show that LRP16 integrates into the NF-κB transcriptional enhanceosome through associating with its p65 component and demonstrated its crucial role for NF-κB activation inside the nucleus. In addition, a positive link between the expression intensity of LRP16 in the nuclei and the NF-κB activity in human gastric carcinoma specimens was preliminarily established. Placing these findings in perspective, LRP16 may represent a therapeutic target in tumors involving excessive NF-κB activity.

## Results

### LRP16 was identified as a novel interactor of NF-κB subunit p65

**Figure 1:**
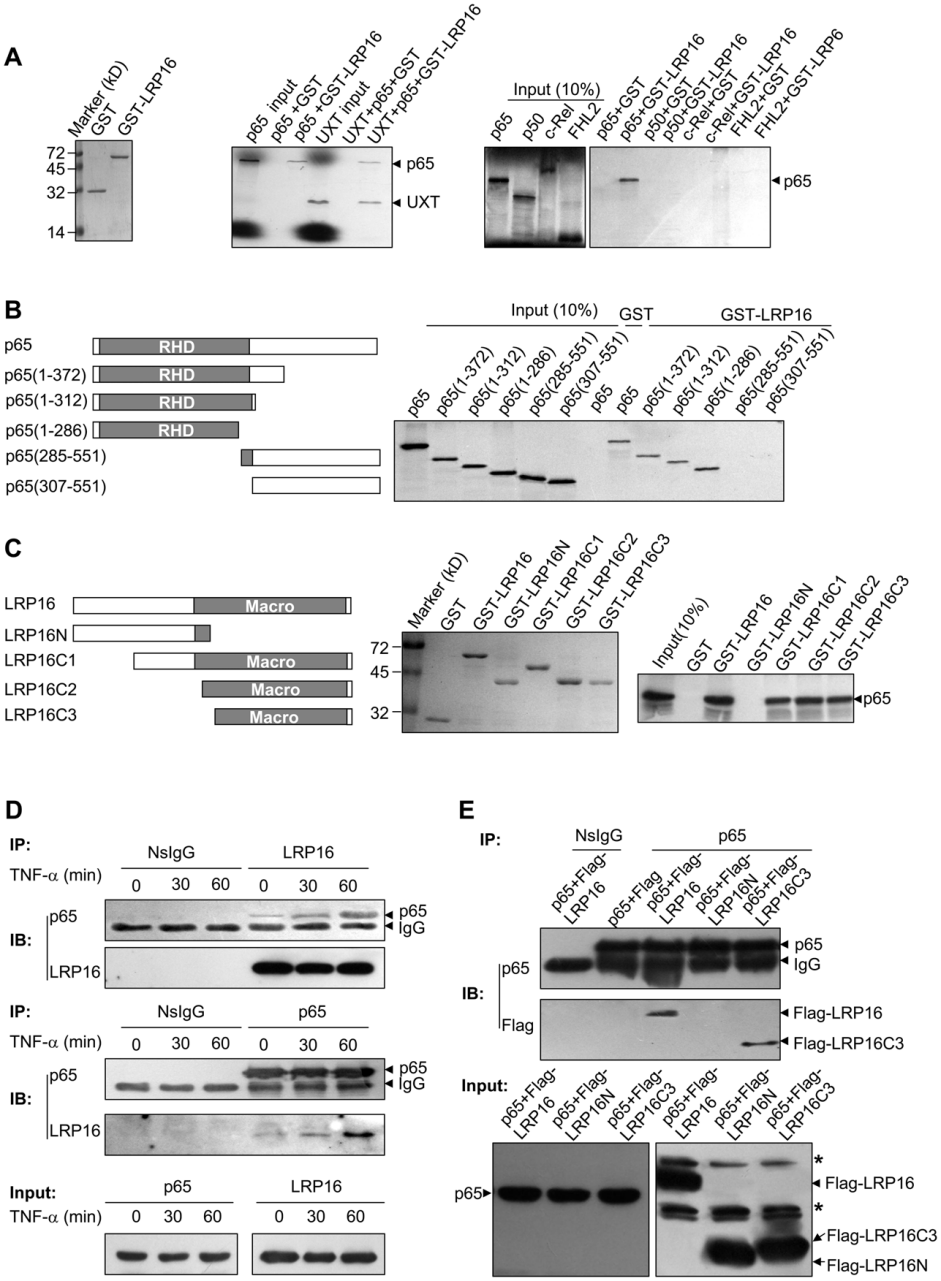
Physical interaction of LRP16 and p65. **(A)** GST and GST-LRP16 stained with Coomassie Blue are shown in the left panel. [^35^S]methionine-labeled UXT, p65, p50 or c-Rel was incubated with GST or GST-LRP16 as indicated in the middle and right panels. **(B)** The left panel shows a schematic illustration of p65 and its mutants. GST pull-down assays were performed with various [^35^S]-labeled mutants of p65 and GST-LRP16 or GST alone (right panel). **(C)** The left panel shows a schematic illustration of LRP16 and its mutants. The middle panel shows GST, GST-LRP16 and its mutants stained with Coomassie Blue. GST pull-down assays were performed with [^35^S]-labeled p65 and GST-LRP16 mutants or GST alone (right panel). **(D)** 293T cells were treated with TNF-α (10 ng/ml) for the indicated times. Cell lysates were immunoprecipitated and immunoblotted with the indicated antibodies. **(E)** 293T cells were cotransfected with p65 and Flag-tagged constructs. Forty-eight hours after transfection, cells were lysed and the lysates subjected to CoIP using the indicated antibodies. The asterisk indicates a non-specific band. NsIgG: non-specific IgG.

Previously, we identified the physical interaction of LRP16 with UXT [23], which had been reported to interact with several NF-κB members including p65, p50 and c-Rel [26]. We proposed that LRP16 may participate in the NF-κB transcriptional complex. To validate this hypothesis, we assessed interactions among LRP16, UXT, and NF-κB members using pull-down assays. As shown in Figure 1A, p65 and UXT were both pulled down by GST-LRP16. However, neither p50 nor c-Rel was pulled down by GST-LRP16 (Figure 1A). To further explore which region was necessary for p65-LRP16 association, we used a series of p65 and LRP16 deletion mutants in GST pull-down assays. The p65 fragments that spanned amino acids 1–286, 1–312, and 1–372, but not the fragments that spanned amino acids 285–551 and 307–551, were pulled down by GST-LRP16 (Figure 1B). Full-length p65 was pulled down by GST-LRP16-C1, GST-LRP16-C2, and GST-LRP16-C3, but not by GST-LRP16-N (Figure 1C). These results suggest that LRP16 can interact with p65 in a cell-free system. Moreover, this interaction appears to be mediated primarily by the macro domain of LRP16 and a specific region of the Rel homology domain (RHD) harboring in p65 rather than in p50 and c-Rel.

To confirm that LRP16 can interact with p65 in cells, we performed coimmunoprecipitation (CoIP) experiments using 293T cells that had or had not been treated with TNF-α. In the absence of TNF-α stimulation, only a weak band that corresponded to endogenous LRP16 in the complexes immunoprecipated with anti-p65 antibody was detected. However, stronger bands were detected during a time course of TNF-α treatment. Similarly, reciprocal results were obtained by using anti-LRP16 antibody (Figure 1D). Next, p65 was cotransfected with Flag-tagged LRP16, LRP16N or LRP16C3, and CoIP assays were subsequently performed using the antibodies indicated (Figure 1E). Consistent with the results from the GST pull-down assays (Figure 1C), p65 was able to interact with the full-length LRP16 and LRP16C3, but not LRP16N, in cells. Immunofluorescence analysis revealed that LRP16 and p65 predominantly localized in the nucleus and cytoplasm, respectively, in the absence of TNF-α treatment. However, they colocalized in the nucleus when cells were stimulated with TNF-α (Figure S1A). These results suggest that LRP16 may participate in the NF-κB transcriptional complex through associating with p65 after the nuclear translocation of NF-κB.

### Overexpression of LRP16 does not significantly alter the endogenous NF-κB activity

**Figure 2:**
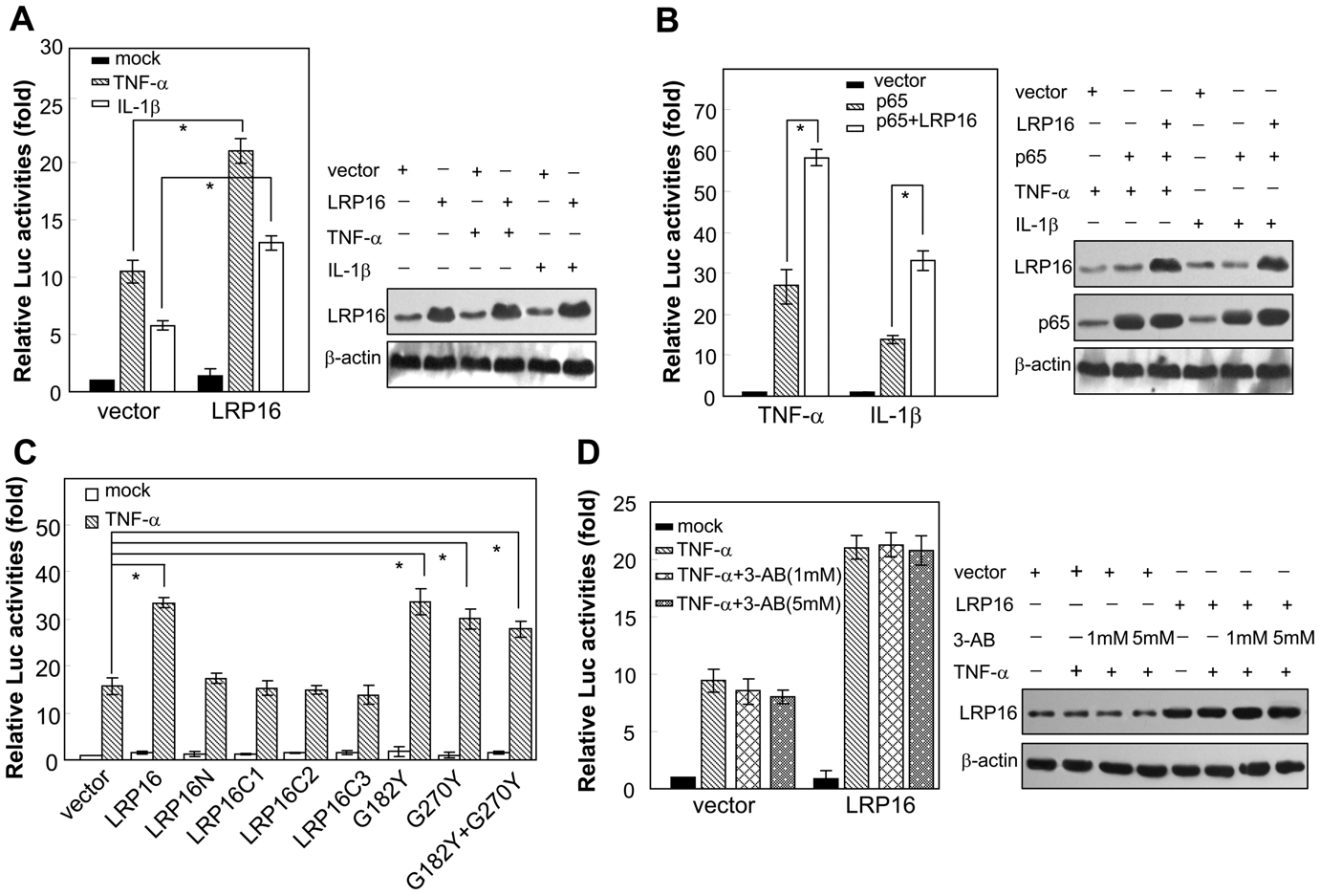
Ectopic expression of LRP16 markedly upregulates cytokine-induced κB-luc activity. **(A–D)** 293T cells were cotransfected with 3×κB-luc and the indicated vectors. Forty-two hours after transfection, cells were simulated with 10 ng/ml TNF-α or 20 ng/ml IL-1βfor 7 h before luciferase assays were performed. The relative levels of luciferase activity were normalized to the activity obtained after cotransfection of 3×κB-luc and the empty expression vector, which was arbitrarily assigned a value of 1. All experiments were performed in triplicate and were repeated at least three times, and the results are expressed as means±SD. ^*^ *P*<0.05.

To investigate the functional significance of LRP16-p65 interaction, we used a κB-luc reporter gene initially to evaluate the regulatory effect of LRP16 on the activation of NF-κB. The results revealed that the ectopic expression of LRP16 increased TNF-α- or IL-1β-induced κB-luc activity by more than 1.5 fold (Figure 2A). Gene activation induced by p65 alone was also increased in cells in which LRP16 was overexpressed (Figure 2B). Similar results were observed in MCF-7 and HeLa cells (). In addition, we also found that the GAL4BD-p65 induction of GAL4-luc activity was further markedly enhanced by LRP16 (Figure S3). Next, several LRP16 mutants, including G182Y, G270Y, and G182Y plus G270Y, which had been reported to efficiently block the mono-ADP-ribose binding capacity of LRP16 [24], and LRP16N, LRP16C1, LRP16C2, and LRP16C3, were compared with the wild-type LRP16. As expected, the LRP16N, which did not associate with p65, failed to enhance reporter gene activity relative to the empty vector control. Unexpectedly, all the mutants with N-terminal deletion also failed to enhance the activity of the reporter gene; however, the mutants G182Y, G270Y, and G182Y plus G270Y augmented κB-luc activity after TNF-α treatment to nearly the same extent as the full-length LRP16 did (Figure 2C). These results suggest that the N- and C-terminal portions of LRP16 are both required to coactivate NF-κB; however, its capacity to bind mono(ADP-ribose) is not required.

An important biochemical characteristic of LRP16 is its ability to senses PAR synthesis, which enables LRP16 to be recruited to PARP-1 targeted DNA [20]. To determine whether the coactivation of NF-κB by LRP16 in response to TNF-α is linked to its ability to senses PAR synthesis, we carried out luciferase assays after we had added 3-aminobenzamide (3-AB), a potent enzyme activity inhibitor of PARP-1, to the culture medium. As shown in Figure 2D, we did not observe significant inhibition by 3-AB on LRP16-enhanced reporter gene activity.

**Figure 3:**
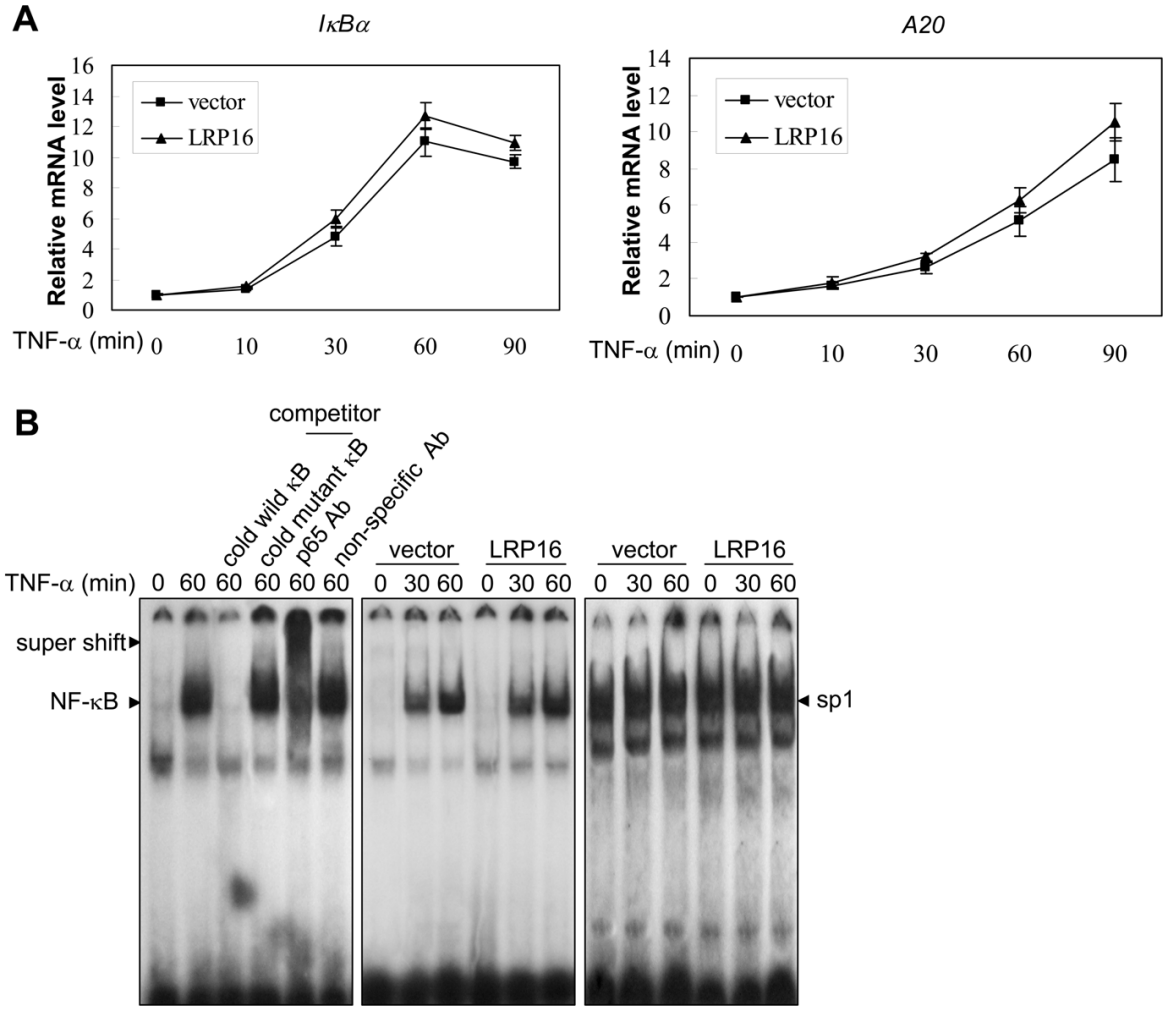
Ectopic expression of LRP16 does not significantly augment TNF-α-induced expression of the endogenous NF-κB target genes and NF-κB DNA binding capacity. (**A**) 293T cells, which were stably transfected with the LRP16 expression construct or empty vector, were treated with TNF-α (10 ng/ml) for the indicated times. Endogenous expression of *IκBα* and *A20* mRNA was measured by qPCR. Data represent means±SD (error bars) of at least three independent experiments. (**B**) 293T cells, which were stably transfected with the LRP16 expression or empty vector, were treated with TNF-α (10 ng/ml) for the indicated times, lysed and the nuclear extracts subjected to EMSA assays. Sp1 was used as a loading control.

To test whether LRP16 had any measurable effects on inducible genes that are regulated by NF-κB such as *Iκ* *Bα* or *A20* [27], [28], we performed qPCR experiments in 293T cells with or without stable LRP16 transfection. As shown in Figure 3A, ectopic LRP16 expression in 293T cells only slightly increased the mRNA levels of *Iκ* *Bα* and *A20* within 90 minutes after TNF-α treatment. We also measured the mRNA levels of *Iκ* *Bα* and *A20* at 4- and 6-hours after TNF-α treatment, but no significant difference was observed between LRP16-transfected and mock-transfected cells. Next, we analyzed the endogenous NF-κB DNA binding activity in 293T cells with stable LRP16 transfection by performing electrophoretic mobility shift assays (EMSA). As shown in Figure 3B, there was no detectable NF-κB/DNA binding band in the absence of TNF-α treatment. LRP16 overexpression did not also induce any detectable basal NF-κB binding activity. In the presence of TNF-α stimulation, robust NF-κB binding band was detected; however, this band was not further strengthened by ectopic expression of LRP16 in cells.

### Knockdown of LRP16 not only attenuates NF-κB transcriptional activity but also it's DNA binding capacity

**Figure 4:**
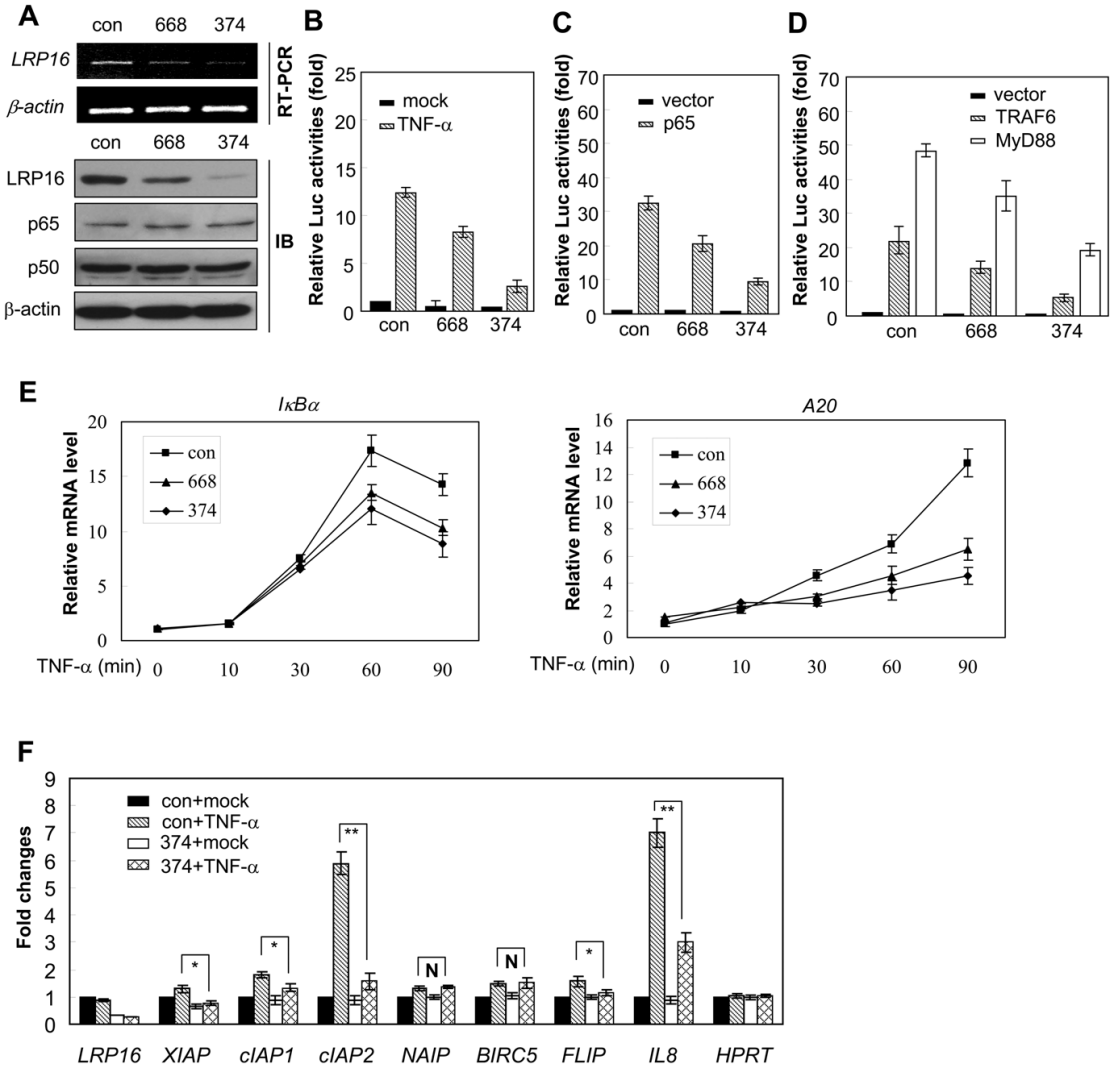
Knockdown of LRP16 markedly attenuates NF-κB transcriptional activity. (**A**) 293T cells were transfected with the indicated siRNAs. Forty-eight hours after transfection, the endogenous expression of LRP16 mRNA was monitored by RT-PCR, and the protein levels of LRP16, p65, and p50 were determined by immunoblotting. β-actin was used as a loading control. (**B–D**) 293T cells were cotransfected with 3×κB-luc, siRNA and vectors as indicated. Forty-two hours after transfection, cells were stimulated with 10 ng/ml TNF-α for 7 h and then luciferase assays were performed. The relative levels of luciferase activity were normalized to the activity obtained after cotransfection of 3×κB-luc and the empty expression vector, which was arbitrarily assigned a value of 1. All experiments were performed in triplicate and were repeated at least three times, and the results are expressed as means±SD. (**E**) 293T cells were transfected with the indicated siRNAs. Forty-eight hours after transfection, cells were stimulated with 10 ng/ml TNF-α for the indicated times. Endogenous expression of the indicated genes was measured by qPCR. Data represent means±SD (error bars) of at least three independent experiments. (**F**) 293T cells were treated as in (E), cells were stimulated with 10 ng/ml TNF-α for 90 min. Endogenous expression of the indicated genes was measured by qPCR. Data represent means±SD (error bars) of at least three independent experiments. *P<0.05, **P <0.01, ^N^ *P*>0.05.

To further verify whether LRP16 is required for the transcriptional activation of NF-κB, we examined the κB-luc activity after LRP16 had been knocked down via RNAi. Compared with the control siRNA, both siRNAs against LRP16 (374 and 668) caused a specific reduction of LRP16 expression at both the mRNA and protein levels in 293T cells but did not affect the expression of the *p65*, *p50*, and *β*-*actin* genes (Figure 4A). Consistent with our previous report [22], [23], 374 reproducibly gave better knockdown than 668 and therefore 374 was used more frequently in later experiments. As shown in Figure 4B, knockdown of the endogenous LRP16 dramatically inhibited TNF-α-induced reporter gene activity. The activation of κB-luc induced by p65 alone was also markedly attenuated in LRP16-deficient 293T cells (Figure 4C). Similar results were observed in HeLa and MCF-7 cells (Figure S4). Ectopic expression of MyD88 and TRAF6, which are well known to induce NF-κB activity [29], [30], did not effectively rescue TNF-α-stimulated κB-luc activities in LRP16-knockdown 293T cells (Figure 4D).

We next investigated how the induction of NF-κB-dependent genes was influenced by knocking down endogenous LRP16 expression. In this case, the induction of *IκBα* and *A20* was markedly attenuated (Figure 4E). In addition, the TNF-α induction of several other NF-κB-dependent genes including *XIAP*, *cIAP1*, *cIAP2*, *FLIP*, and *IL8*, but not *NAIP* and *BIRC5*, were also markedly attenuated in LRP16-deficient 293T cells (Figure 4F). Collectively, our data strongly indicate that the expression of a certain level of LRP16 is necessary for the induction of genes that are tightly regulated by NF-κB.

**Figure 5:**
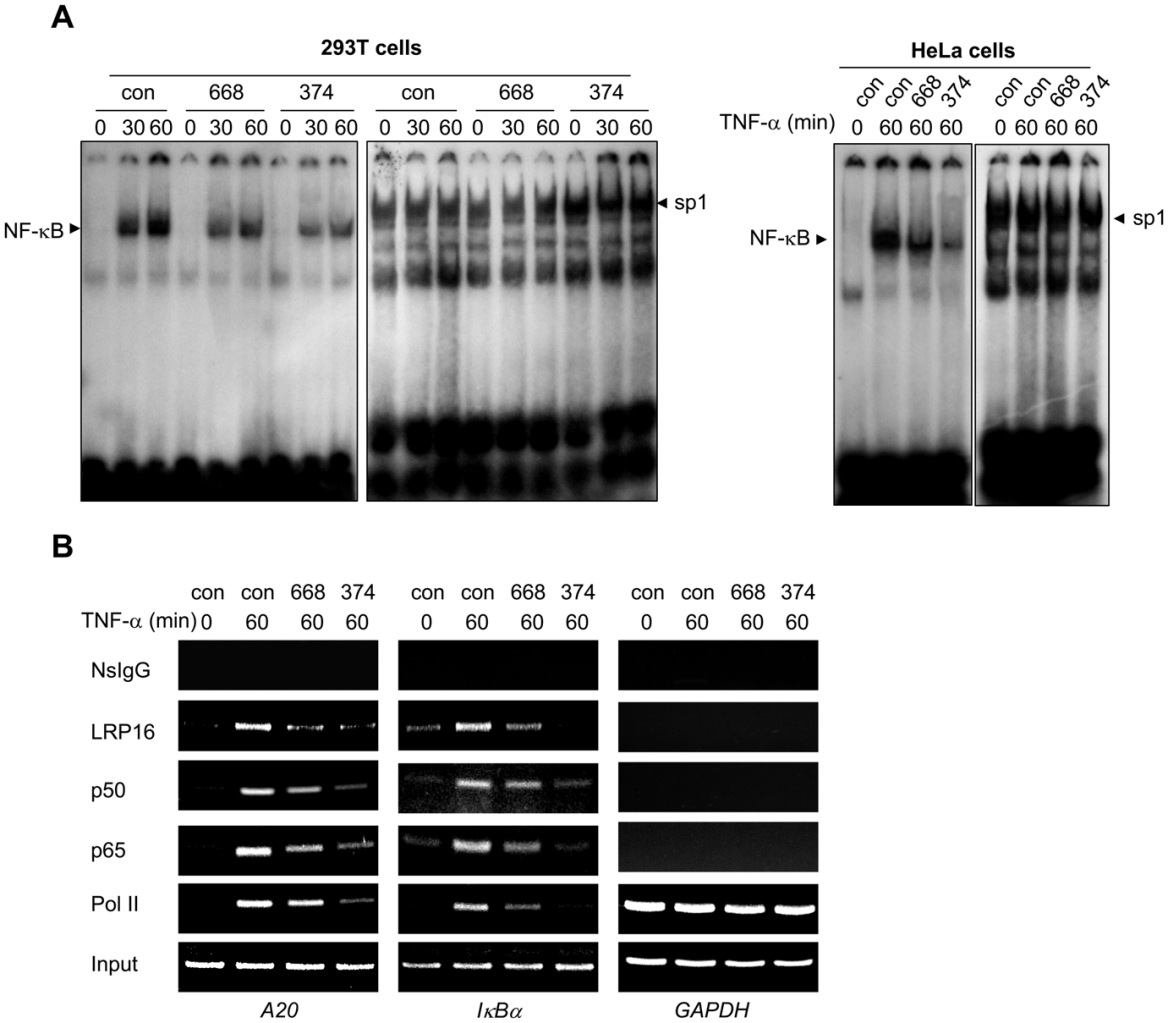
Knockdown of LRP16 sharply attenuates the DNA binding capacity of NF-κB. (**A**) 293T or HeLa cells were transfected with the indicated siRNAs. Forty-eight hours after transfection, cells were stimulated with 10 ng/ml TNF-α for the indicated times, lysed and the nuclear extracts subjected to EMSA assays. Sp1 was used as a loading control. (**B**) 293T cells were treated as in (A). The ChIP assays were performed for the *A20*, *IκBα* and *GAPDH* promoters using the indicated antibodies and corresponding primers described in Materials and Methods.

To further confirm whether LRP16 is required for the activation of NF-κB, we analyzed the binding activity of NF-κB with its cognate DNA sequence in LRP16-knockdown 293T and HeLa cells by performing EMSA. Compared with that from control-siRNA-transfected cells, the TNF-α-stimulated DNA binding capacity of NF-κB was significantly diminished in nuclear extracts from LRP16-specific knockdown cells (Figure 5A). In addition, we also observed that this diminish was correlated with the effectiveness of the siRNAs administered.

To substantiate this finding, we took advantage of chromatin immunoprecipitation (ChIP) assays to test whether the binding of NF-κB to its endogenous promoter was influenced in LRP16-knockdown cells. As shown in Figure 5B, TNF-α not only stimulated a remarkable recruitment of p65, p50 and RNA polymerase II, but also LRP16 on both *A20* and *IκBα* promoters in control-siRNA transfected cells, strongly indicating that LRP16 is a component of NF-κB enhanceosome. However, knockdown of the endogenous LRP16 led to a considerable decrease in the amount of p65 associated with its cognate promoters. In addition, the amount of both NF-κB p50 and RNA polymerase II at their cognate targets was also dramatically decreased in LRP16-deficient cells. As a negative control, knockdown of LRP16 had no effect on the GAPDH transcriptional complex. These finding indicate that LRP16 is an essential component of NF-κB enhanceosome.

### Knockdown of LRP16 blunts the interaction of NF-κB with p300/CBP/CARM1 in nuclei

The activation of NF-κB involves a series of molecular events in the cytoplasm and in the nucleus [3]. To rule out the possibility that LRP16 might act on the TNF-α-induced nuclear translocation of NF-κB, we examined the effects of LRP16 knockdown on IKK activity and the expression of IκBα protein induced by TNF-α over time. The results showed that the siRNAs targeting LRP16 did not affect TNF-α-induced IKK activities (Figure S5A). In addition, IκBα level in 293T cells was rapidly and transiently reduced upon TNF-α stimulation, whether or not the endogenous LRP16 had been inhibited (Figure S5B). By immunofluorescence analyses, we did not observe marked difference for the nuclear translocation of p65 between the LRP16-deficient and mock-transfected cells upon TNF-α stimulation (Figure S1B and S1C). These results demonstrate that LRP16 performed its function via targeting NF-κB itself in the nucleus, but not via affecting IκBα degradation and NF-κB nuclear translocation. This conclusion was further authenticated by measuring the amount of p65 in nuclei after TNF-α treatment (Figure S5C).

**Figure 6:**
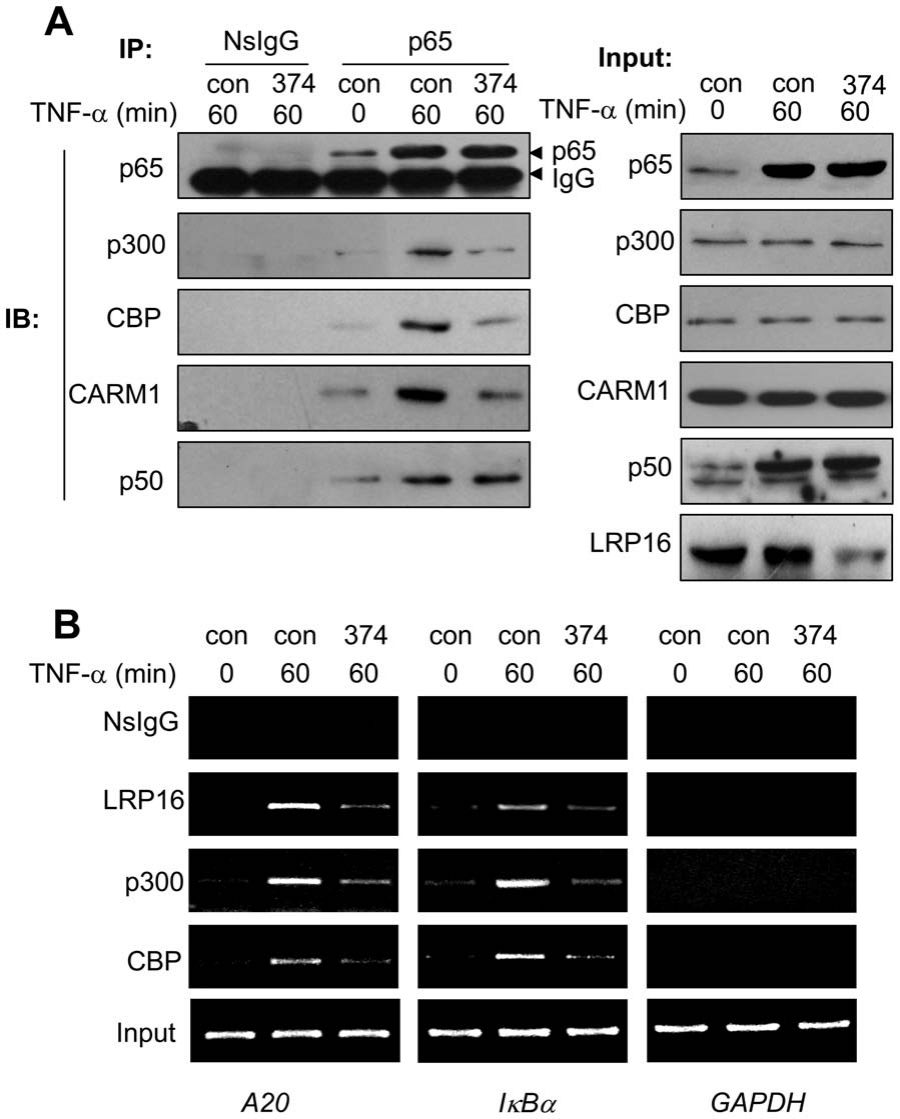
Knockdown of LRP16 impairs the formation or stability of NF-κB/p300/CBP/CARM1 complex inside the nucleus. (**A**) 293T cells were transfected with the indicated siRNAs. Forty-eight hours after transfection, cells were treated with 10 ng/ml TNF-α for the indicated times and the nuclear fraction was purified. Equal amounts of nuclear lysates were immunoprecipitated and immunoblotted with the indicated antibodies. (**B**) 293T cells were treated as in (A), ChIP assays were performed for the *A20*, *IκBα* and *GAPDH* promoters using the indicated antibodies and corresponding primers described in Materials and Methods.

Due to the lack of domains in LRP16 that are found in conventional transcriptional coregulators, we speculated that LRP16 may execute its coactivation effect on NF-κB by affecting the assembly or stability of the NF-κB transcriptional complex in nucleus. To validate this hypothesis, we analyzed the interactions of NF-κB with its coactivators present in the nucleus, including p300, CBP, and CARM-1, by performing CoIP experiments. As shown in Figure 6A, by using p65/p50 interaction as a control, we observed that the association of p65 with these coactivators in the presence of TNF-α was considerably reduced in LRP16-knockdown cells. In addition, we also observed the decreased recruitment of p300/CBP on the promoters of both *A20* and *IκBα* in LRP16-knockdown cells (Figure 6B). These results demonstrate that knockdown of LRP16 blunts the interactions of NF-κB with other essential coactivatiors such as p300/CBP.

### Knockdown of LRP16 sensitizes TNF-α-induced cell apoptosis

**Figure 7:**
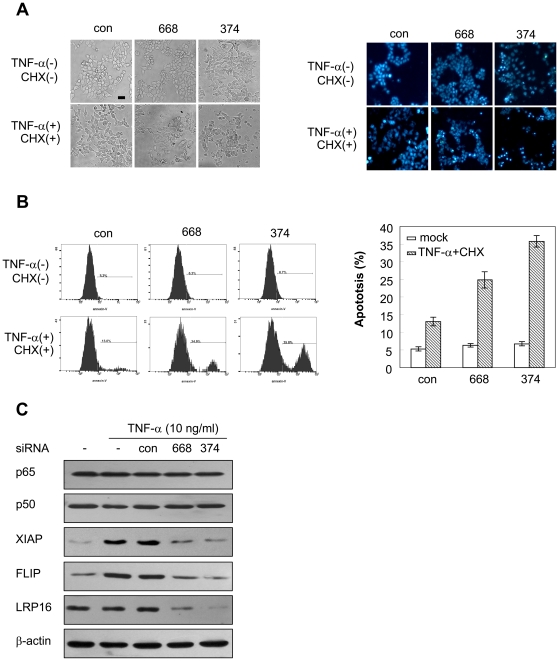
Knockdown of LRP16 sensitizes TNF-α-induced cell apoptosis. (**A**) 293T cells were transfected with the indicated siRNAs. Forty-eight hours later, cells were treated with TNF-α plus CHX or left untreated for 22 h. Representative microscopic images are shown (right panel for DAPI staining). Bar = 20 µm. (**B**) 293T cells were treated as in (A), the percentage of apoptotic cells was monitored by annexin V staining followed by FACS analysis. Experiments to analyze apoptosis were performed in triplicate and were repeated at least three times, and the results are expressed as means±SD. (**C**) 293T cells were transfected with the indicated siRNAs. Forty-eight hours after transfection, cells were stimulated with 10 ng/ml TNF-α for an additional 12 h. Total protein was prepared and immunoblotting was performed with the indicated antibodies.

Activation of NF-κB can prevent cell apoptosis induced by TNF-α by promoting the expression of several antiapoptotic genes. A low concentration of cycloheximide (CHX, <10 µg/ml), which is a potent inhibitor of protein synthesis, can dramatically augment TNF-α-induced apoptosis effect [31]. Based on the above-mentioned findings that LRP16 is required for NF-κB activation, we further explored whether TNF-α-induced cell apoptosis is modulated by LRP16 by using TNF-α-sensitive 293T and HeLa cells. The apoptotic rate of both 293T and HeLa cells after treatment with TNF-α plus CHX was not markedly increased by stably transfecting LRP16 or its mutants into these cells (Figure S6). However, when the endogenous LRP16 was inhibited by siRNAs, apoptotic bodies and other dramatic morphological changes indicative of cells undergoing apoptosis were observed under microscope (Figure 7A and S7). Changes of apoptotic percentage quantitatively evaluated by annexin V staining and FASC analysis also revealed that knockdown of LRP16 expression significantly augmented cell apoptosis induced by TNF-α plus CHX (Figure 7B). Similar results were observed in HeLa cells (data not shown). Moreover, we observed that TNF-α-induced upregulation of antiapoptotic protein XIAP and FLIP was efficiently blocked by inhibiting LRP16 expression in 293T cells (Figure 7C).

### Prominent nuclear staining of LRP16 is positively correlated with elevated NF-κB activity in gastric carcinoma samples

**Figure 8:**
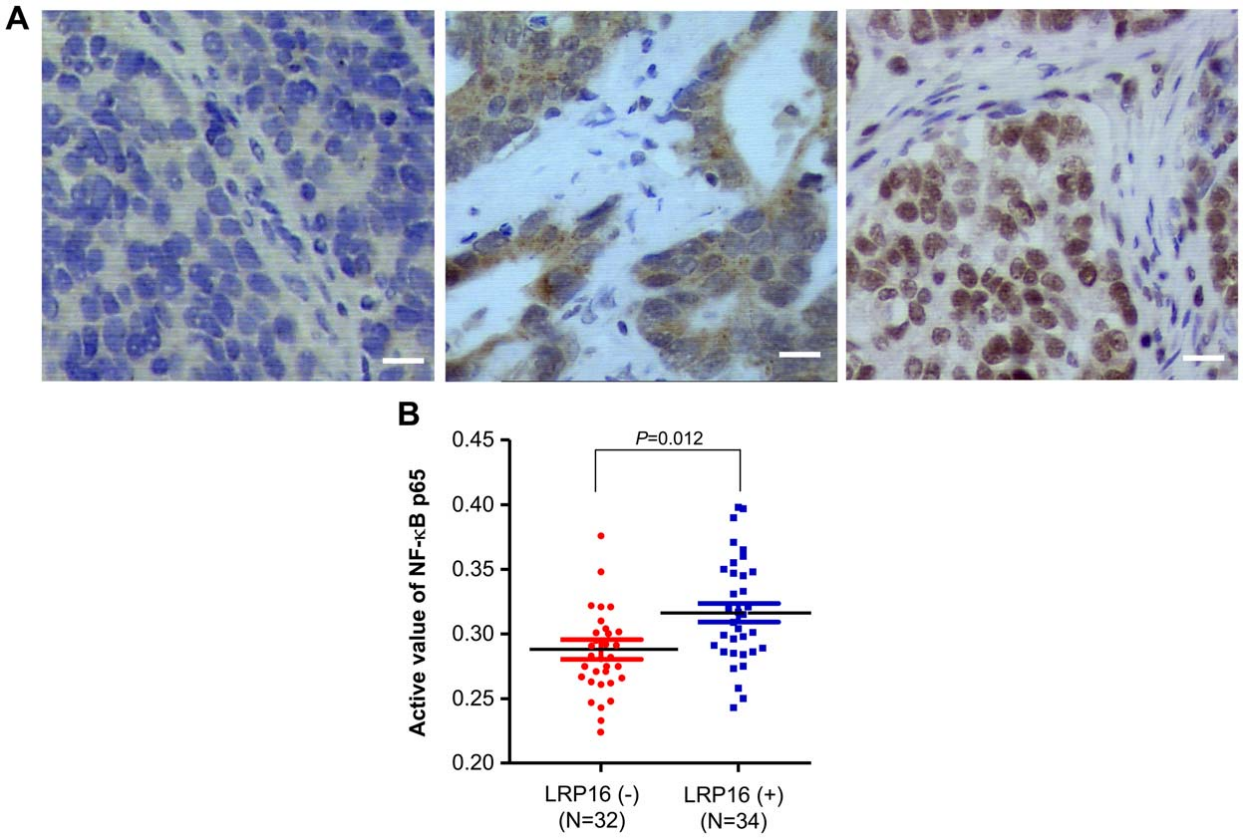
The expression level of LRP16 in nuclei is positively associated with the elevated NF-κB activity in human gastric carcinoma specimens. (**A**) Immunostaining of LRP16 in three representative gastric carcinoma samples. LRP16 is stained dark brown; nuclei were counterstained with hematoxylin (light blue). The left panel shows no staining. Predominant cytoplasmic staining of LRP16 is shown in the middle panel. The right panel shows predominant nuclear staining of LRP16. Bar = 20 µm. (**B**) The DNA binding activity of NF-κB in the LRP16 negative and positive groups was measured by using an ELISA-based chemiluminescent kit as described in Materials and Methods.

Our previous studies revealed that LRP16 can interact with both ERα and AR [22], [23]. In this study, we determined that LRP16 can also associate with the NF-κB transcriptional complex. To clearly determine whether LRP16 expression could be extended to NF-κB activity in clinical samples, human gastric carcinoma specimens were selected, instead of hormone related cancers, in order to exclude the possible competitive interaction interference of LRP16 with other transcription factors, such as ERα and AR. Samples from 66 cases of human gastric carcinoma were initially subject to immunoblotting analysis of total LRP16 protein amount and enzyme-linked immunosorbent assay (ELISA)-based evaluation of active NF-κB. However, this assay did not significantly link the LRP16 expression level with NF-κB activity (data not shown). Then, we turned to perform immunohistochemical staining of LRP16 in these samples. Photomicrographs from three selected cases are presented in Figure 8A. Negative nuclear (left panel) and prominent cytoplasmic staining (middle panel) of LRP16 were defined as negative, and prominent nuclear staining (right panel) was defined as positive, as described previously [32]. Under these criteria, 32 and 34 cases were classified as LRP16 negative and LRP16 positive, respectively. The level of active NF-κB in the LRP16-positive subset (OD value = 0.316±0.042) was significantly higher than that in the LRP16-negative subset (OD value = 0.288±0.036) (*t* = 31.64; *P* = 0.012) (Figure 8B). The correlation between activity of NF-κB and LRP16 expression was statistically significant for these two patient subsets (*r* = 0.326; *P* = 0.014). These data suggest that the prominent nuclear distribution of LRP16 may be positively correlated with the increased NF-κB activity in human gastric carcinomas and further support the notion that LRP16 is a critical regulator for NF-κB activation inside the nucleus.

## Discussion

In this study, the macro domain protein LRP16 was identified as a novel interactor of NF-κB component p65. By a series of independent approaches, we demonstrated that LRP16 participates in the NF-κB enhanceosome and is crucial for the recruitment of NF-κB for essential coactivators such as p300 and CBP. Knockdown of LRP16 led to impaired NF-κB activity and signaling output. The regulatory role of LRP16 on NF-κB activity was further corroborated by a positive relationship between the staining intensity of LRP16 in the nuclei of tumor cells and the level of NF-κB activity in human gastric carcinomas. These findings highlight an important nuclear function of LRP16 in the regulation of NF-κB transcriptional activity and suggest that LRP16 might be an important contributor to elevated NF-κB activity in tumors.

The biochemical feature of binding PAR is required for macro domain proteins including LRP16 to participate into PARP-1-based complex in response to DNA damage [19], [20]. In this study, the luciferase results as shown in Figure 2C and 2D did not demonstrate the link between the LRP16 coactivation of TNF-α-induced NF-κB transcriptional activity and its biochemical feature of binding ADP-ribose metabolites. These results suggest that the biochemical activity of binding ADP-ribose metabolites is dispensable for macro domain proteins when they serve as transcriptional cofactors.

By knockdown assays, we demonstrated that LRP16 is essential for NF-κB activation. In addition, this interference also sensitizes TNF-α-induced cell apoptosis. Although we also demonstrated that the ectopic expression of LRP16 can significantly increase TNF-α-induced κB-luc activity, this ectopic expression in 293T cells did not further enhance TNF-α-induced NF-κB-dependent gene expression, and its DNA binding ability. These results indicate that the endogenous amount of LRP16 inside the nuclei of some cell types is in a saturate status for maximally activating the endogenous NF-κB activity. However, exogenous LRP16 is required for maximally activate the exogenous κB-luc activity.

Coactivators are frequently observed to be shared by different transcription factors, but the differential requirement for shared coactivators may occur in these cases [33], [34]. In addition to NF-κB, LRP16 can interact with some steroid receptors such as ERα and AR, and is essential for their transactivation [22], [23]. The macro domain alone of LRP16 is enough to mediate its interaction with both p65 and AR. The macro domain alone can augment AR-dependent gene transcription in the same way as the wild type protein [23]; however, the macro domain and the non-macro domain regions of LRP16 are both required to activate NF-κB-dependent transcription activity.

Although regulatory events in the nucleus all shape the strength and duration of the NF-κB transcriptional response, the different factors recruited by NF-κB posses differential action mechanisms. p300/CBP was found to associate directly with NF-κB and form a bridge between NF-κB and the basal transcriptional machinery [11], [12]. CARM-1 synergistically coactivates NF-κB-mediated transcription in a promoter-dependent manner, in concert with p300/CBP and p160 [13]. In this study, we demonstrated that the nuclear translocation of NF-κB in the presence of TNF-α stimulation was not affected by LRP16 knockdown. However, the formation or stability of NF-κB transcription complex (NF-κB/p300/CBP/CARM1) was markedly impaired in nuclei of LRP16-deficient cells. Our findings support the notion that LRP16 is essential for the assembly or stability of the functional NF-κB transcription complex in the nucleus. The precise mechanism regarding how LRP16 affects the formation of functional NF-κB complex still remains to be studied and is an interesting and important subject of further investigation.

NF-κB-associated pathways have been widely implicated in oncogenesis and tumor progression by stimulating cell proliferation, inhibiting apoptosis, and promoting metastasis and angiogenesis [35]. Aberrant activation of NF-κB is frequently observed in several tumor types including gastric carcinomas [36], [37], [38]. In this study, a positive link between the prominent staining of LRP16 inside the nucleus and the elevated NF-κB activity was established in gastric carcinoma specimens. In view of the fact that the clinicopathological data we obtained previously from a large number of samples, which included breast cancer, gastric cancer and colorectal carcinoma, indicated that predominant nuclear staining of LRP16 in tumor cells is significantly linked to high-risk prognostic indices and short survival term [32], [39], [40], we proposed that the aberrant nuclear accumulation of LRP16 would be a common mechanism employed by tumor cells by which NF-κB was excessively activated. The predominant nuclear staining of LRP16 in tumor cells may be a valuable indicator for the evaluation of excessive NF-κB activity.

Aberrant stimulation of estrogen and androgen plays a central role in the genesis and progression of breast cancer and prostate cancer, respectively [41], [42]. Crosstalk or reciprocal regulation between hormone-dependent pathways, including ERα- and AR-mediated signal transduction, and NF-κB signaling has been extensively established at multiple signaling nodes [43], [44], [45]. However, reasonable explanations for excessive NF-κB activity in hormone-dependent cancers remain to be investigated [46], [47], [48]. Previously, we demonstrated that estrogen can upregulate LRP16 expression in estrogen-dependent breast cancer, endometrial cancer, and ovary cancer cell lines [22], [25], [49], [50], [51], [52]. Androgen can also upregulate LRP16 expression in androgen-sensitive prostate cancer cell lines [23]. The expression level of LRP16 in hormone-dependent cancer cell lines is dependent on hormone action. In addition, we also previously revealed the promotion of LRP16 to proliferation or invasive growth of hormone-dependent cells [22], [23], [25], [51]. The identification of the hormone-responsive gene LRP16 acting as an crucial coactivator of NF-κB in this study may provide a novel clue for linking hormone-dependent signaling and NF-κB activity in hormone-dependent cancers.

In conclusion, these findings provide definite evidence to support the integration of the macro domain protein LRP16 into the NF-κB transcriptional complex via interaction with p65 and its crucial role for NF-κB activation inside the nucleus. Aberrant nuclear accumulation of LRP16 in tumor cells might represent a therapeutic target for the control of excessive NF-κB activity.

## Materials and Methods

### Reagents

The LRP16-specific siRNAs 374 and 668 and the control siRNA were chemically synthesized by GenChem Co., Ltd (Shanghai, China). TNF-α and IL-1β were purchased from R&D systems. Lipofectamine 2000 was purchased from Invitrogen. SuperFect transfection reagent was obtained from QIAGEN. Antibodies against p65, p50, IκBα, FLIP, CARM1, Sp1, and β-actin were purchased from Santa Cruz Biotechnology. Antibodies against p300/CBP and CARM1 were purchased from Bioworld Technology Co., Ltd. The antibody against XIAP was purchased from Marine Biological Laboratory. The LRP16 antibody was described previously [23]. 3-aminobenzamide (3-AB), cycloheximide (CHX) and the antibody against FLAG tag and RNA polymerase II were obtained from Sigma-Aldrich.

### Plasmids

The LRP16 and UXT constructs were described in our previous reports [23]. The expression vectors for p50, c-Rel, p65 and its mutants, TRAF6, and MyD88, and the luciferase reporter 3×κB-luc, were kindly donated by Professor Chen Wang (Chinese Academy of Science). The mutants of LRP16, including G182Y, G270Y, and G182Y plus G270Y, were provided by Dr. Tero Ahola (Institute of Biotechnology, University of Helsinki). GAL4-luc was prepared by cloning five GAL4 binding sites into the pGL-Basic vector (Promega). The plasmid encoding GAL4BD-p65 was constructed by cloning p65 in-frame with amino acids 1-147 of the GAL4 protein in a pcDNA3.1 (Invitrogen) backbone.

### Human samples

Studies using human tissues were approved by the Institutional Ethical Committee in Chinese PLA General Hospital. Tissues samples of gastric carcinomas were collected from 66 patients who had undergone subtotal gastrectomy in the Chinese PLA General Hospital from August to December in 2009 following written informed consent. The cancerous tissue samples were immediately frozen into liquid nitrogen or fixed in 10% formalin for subsequent analysis. Sixty-six patients were classified into the following groups according to their pathological diagnoses: well-moderately differentiated adenocarcinoma (N = 20), mucinous adenocarcinoma (N = 7), poorly differentiated adenocarcinoma (N = 28), signet-ring cell carcinoma (N = 7), and other gastric carcinoma (N = 4).

### Cell culture, transfection and preparation of nuclear extracts

293T and HeLa cells were obtained originally from the American Type Culture Collection (ATCC, Rockville, MD, USA) and cultured according to the instructions provided. SuperFect reagent was used for DNA transfection, whereas Lipofectamine 2000 was used to transfect cells with siRNA duplexes. Total or nuclear proteins were extracted using a ReadyPrep™ protein extraction kit (Bio-Rad Laboratories).

### In vitro transcription/translation and GST pull-down assays

GST and GST fusion proteins were prepared as described previously [23], [53]. ^35^S-labeled proteins were produced by using a TNT-coupled in vitro transcription and translation (IVT) system (Promega), with the expression vectors for p65 and its derivatives, p50 and c-Rel in pcDNA3 or pcDNA3.1. GST pull-down assays were performed primarily as described previously [23], [53].

### Coimmunoprecipitation (CoIP) and immunoblotting

Cells were cultured in 10-cm dishes, transfected with expression vectors or siRNA duplexes or mock transfected, and then were treated with TNF-α for the indicated times. The cells were then harvested and total protein or nuclear proteins were extracted. The CoIP and immunoblotting were performed as described previously [23], [53].

### Luciferase assays

Cells were seeded in 35-mm culture dishes and were cotransfected with 0.5 µg of κB-luc or GAL4-luc reporter construct and other constructs as indicated, or 1 µg of siRNA duplexes. pRL-SV40 (1 ng) was used as an internal control. The total amount of nucleotide was kept constant by supplementing with pcDNA3. Fourty-two hours later, the cells were treated with the reagents indicated or left untreated. Luciferase activity was analyzed with the Dual-Luciferase Reporter Assay System (Promega) as described previously [23], [53].

### IKK activity analyses

293T cells that had been transfected with the indicated siRNAs were treated with TNF-α (10 ng/ml) or left untreated for 15 min. IKK kinase assays were performed with the IKKα/β assay/Inhibitor Screening Kit (Abnova), in accordance with the manufacturer's instructions.

### Immunofluorescence and microscope

Cells grown on coverslips were fixed with 4% paraformaldehyde, permeabilized in 0.5% TritonX-100, blocked with 10% goat serum, and stained with either mouse-anti LRP16 or rabbit-anti p65 antibodies followed by fluorescein isothiocyanate (FITC)-conjugated goat anti-mouse IgG (Santa Cruz) or Alexa-Fluor 594-conjugated goat anti-rabbit IgG (Invitrogen). Nuclei were counterstained with DAPI. Immunofluorescence analysis was performed using a Nikon fluorescence microscope equipped with the appropriate filters for three-color imaging of cells and with a CDD camera.

### Quantitative RT-PCR (qPCR)

Total RNA was isolated using TRIzol Reagent (Invitrogen). cDNA was prepared using PrimeScript™ reverse transcriptase (Takara Shuzo Co., Tokyo, Japan) and 1 to 2 µg of total RNA. cDNA was subjected to qPCR using the SYBR Green PCR Reagents Kit (Bio-Rad). PCR primers were as follows: *LRP16* sense, 5′-CCGCAGCGACATCACCAAGC-3′, antisense 5′-TCCGGCACTCGTCGGTAAGC-3′. *A20* sense 5′-CCTGCTGGCTGCCTGTCT-3′, antisense, 5′-GAACCTGGACGCTGTGGG-3′; *IκBα* sense, 5′-GAGGAAATACCCCCCTACACC-3′, antisense 5′-AGCACCCAAGGACACCAAAAG-3′; *IL8* sense, 5′-ACTCCAAACCTTTCCACC-3′, antisense 5′-AAACTTCTCCACAACCCTC-3′; *cIAP1* sense, 5′-TCCAGTTCAGCCTGAGCAGC-3′, antisense 5′-TCCCAACACCTCAAGCCACC-3′; *cIAP2* sense, 5′-TTTTGCTGTGATGGTGGACTC-3′, antisense 5′-TCTCCTGGGCTGTCTGATGTG-3′; *XIAP* sense, 5′-CTTCCAAGAAATCCATCCA-3′, antisense, 5′-TTCCAATCAGTTAGCCCTC-3′; *FLIP* sense, 5′-CTCACCTTGTTTCGGACT-3′, antisense, 5′-CCTTGCTTATCTTGCCTC-3′; *NAIP* sense, 5′-TTTGAATGATGACAGCGTG-3′, antisense, 5′-CTGTGAAATGCCTGGAGAT-3′; *BIRC5* sense, 5′-CCAGACTTGGCCCAGTGTTTC-3′, antisense 5′-ACTTTCTCCGCAGTTTCCTCA-3′; *HPRT* sense, 5′-TTGCTCGAGATGTGATGAAAGGA-3′; antisense, 5′-TTCCAGTTAAAGTTGAGAGATCA-3′. Reactions were run on a LightCycler (Roche).

### Electrophoretic mobility shift assay (EMSA)

EMSA was carried out essentially as described previously [50], [53]. Wide-type κB probe and Sp1 probe were labeled with T4 polynucleotide kinase and [γ-^32^P]dATP. For each 25 µl reaction, 10 µg nuclear extracts were incubated with ^32^P-labeled probes in binding buffer for 20 minutes at room temperature. A 100-fold excess of unlabeled wild-type or κB mutant oligonucleotide was used for competition experiments. An amount of 6 µg of anti-p65 or non-specific IgG was used for supershift experiment. The sample was run through a 6% polyacrylamide gel then dried and subjected to autoradiography. The following probes were used: wild-type κB probe (AGTTGAGGGGACTTTCCCAGGC), mutant κB probe (AGTTGAGGCGACTTTCCCAGGC), and Sp1 probe (-ATTCGATCGGGGCGGGGCGAGC).

### Chromatin immunoprecipitation (ChIP) assay

The ChIP assays were carried out essentially as described previously [49], [50]. 293T cells were transfected with the indicated siRNAs (control, 668 and 374). Forty-eight hours later, cells were treated with TNF-α (10 ng/ml) for 60 minutes, then ChIP assays were performed using Chromatin Immunoprecipitation Assay Kit (Millipore Corporation, MA, USA) according to the manufacturer's instructions. The following antibodies were used: anti-LRP16, anti-p65, anti-p50, anti-polymerase II, anti-p300, anti-CBP, and non-specific IgG. DNA fragment were purified with use of a PCR Production Purification Kit (Qiagen). The presence of the target DNA fragments in both the input and the recovered DNA immunocomplexes was detected by PCR. The following primers were used: *A20* promoter sense (5′-CAGCCCGACCCAGAGAGTCAC-3′) and antisense (5′-CGGGCTCCAAGCTCGCTT-3′); *IκBα* promoter sense (5′-TAGTGGCTCATCGCAGGGAG-3′) and antisense (5′-TCAGGCTCGGGGAATTTCC-3′); *GAPDH* promoter sense (5′-AGCTCAGGCCTCAAGACCTT-3′) and antisense (5′-AAGAAGATGCGGCTGACTGT-3′).

### Apoptosis assays

Cells that had been transfected stably with LRP16 or LRP16 mutants or transiently with the indicated siRNA were treated with TNF-α (293T, 50 ng/ml; HeLa, 5 ng/ml) and CHX (5 µg/ml) or left untreated for 22 h. Floating and adherent cells were collected and analyzed using the Annexin V-FITC Apoptosis Detection Kit I (BD Bioscience, CA, USA) in accordance with the manufacturer's instructions. A FACSCalibur flow cytometer (BD Biosciences) was used.

### ELISA-based DNA binding assay for NF-κB p65, and immunohistochemistry (IHC)

The DNA binding activity of NF-κB in the tumor samples was measured by using the enzyme-linked immunosorbent assay (ELISA)-based chemiluminescent NF-κB Binding Assay Kit (Active Motif, CA, USA) in accordance with the manufacturer's instructions.

LRP16 expression in primary tumor tissues was determined by immunohistochemistry (IHC) as described previously [32].

### Statistical analysis

Statistical analyses were performed using SPSS 13.0 for Windows (SPSS Inc, Chicago, Illinois). Paired Student's *t*-tests or two-way ANOVA followed by the student-Newman-Keuls test were used, where applicable, to assess significant differences between groups. To compare NF-κB DNA binding activity between the LRP16 positive group and LRP16 negative group, a *t*-test was used. Spearman's correction test was performed to determine the relationship between the level of expression of LRP16 and the DNA binding activity of NF-κB. *P*<0.05 was considered to be statistically significant.

## Supporting Information

Figure S1 **LRP16 and NF-κB p65 colocalizes in the nuclei of 293T cells in response to TNF-α stimulation. (A–C)** 293T cells were transfected with the indicated siRNAs. Forty-eight hours after transfection, cells were stimulated with 10 ng/ml TNF-α for 1 h, and stained with anti-LRP16 and anti-p65 primary antibodies followed by FITC-conjugated goat anti-mouse IgG and Alexa-Fluor 594 goat anti-rabbit IgG. The nucleus was counterstained with DAPI. Bar = 20 µm.
(PDF)

Figure S2 **Ectopic expression of LRP16 markedly upregulates TNF-α** **-induced κB-luc activity. (A, B)** MCF-7 and HeLa cells were cotransfected with 3×κB-luc and the indicated vectors. Forty-two hours after transfection, cells were simulated with 10 ng/ml TNF-α for 7 h before luciferase assays were performed. The relative levels of luciferase activity were normalized to the activity obtained after cotransfection of 3×κB-luc and the empty expression vector, which was arbitrarily assigned a value of 1. All experiments were performed in triplicate and were repeated at least three times, and the results are expressed as means±SD. *P<0.05.
(PDF)

Figure S3 **Ectopic expression of LRP16 markedly upregulates the GAL4BD-p65 induction of GAL4-luc activity.** Gal4-luc was cotransfected into 293T cells with the indicated vectors. pRL-SV40 was used as transfection control. Forty-two hours after transfection, cells were lysed for luciferase assays. The relative levels of luciferase activity were normalized to the activity obtained after cotransfection of GAL4-luc and the empty expression vector, which was arbitrarily assigned a value of 1. Data represent means ± SD (error bars) of at least three independent experiments. *P<0.05.
(PDF)

Figure S4 **Knockdown of LRP16 markedly attenuates NF-κB transcriptional activity. (A, B)** MCF-7 and HeLa cells were cotransfected with 3×κB-luc, siRNA and vectors as indicated. Forty-two hours after transfection, cells were stimulated with 10 ng/ml TNF-α for 7 h and then luciferase assays were performed. The relative levels of luciferase activity were normalized to the activity obtained after cotransfection of 3×κB-luc and the empty expression vector, which was arbitrarily assigned a value of 1. All experiments were performed in triplicate and were repeated at least three times, and the results are expressed as means±SD.
(PDF)

Figure S5 **Knockdown of endogenous LRP16 expression in 293T cells does not affect TNF-α-stimulated IKK activity and nuclear translocation of NF-κB.** **(A)** 293T cells were transfected with the indicated siRNAs. Forty-eight hours after transfection, cells were treated with 10 ng/ml TNF-α for 15 min, and lysed for IKK activity analysis. **(B)** 293T cells were transfected with the indicated siRNAs, treated with TNF-α for the indicated time points, lysed and the lysates were subjected to immunoblotting with the indicated antibodies. **(C)** 293T cells were transfected with the indicated siRNAs, treated with TNF-α for the indicated time points, nuclear extracts were used for immunoblotting analysis with the indicated antibodies.
(PDF)

Figure S6 **Ectopic expression of LRP16 or LRP16 mutants do not significantly promote TNF-α-induced cell apoptosis.** **(A, B)** 293T and HeLa cells that had been transfected stably with LRP16 or the indicated LRP16 mutants were treated with TNF-α plus CHX or left untreated for 22 h. Annexin V assays were performed to monitor cell apoptosis.
(PDF)

Figure S7 **Knockdown of LRP16 sensitizes TNF-α-induced cell apoptosis.** HeLa cells were transfected with the indicated siRNAs. Forty-eight hours later, cells were treated with TNF-α plus CHX or left untreated for 22 h. The percentage of apoptotic cells was monitored by annexin V staining followed by FACS analysis. Experiments to analyze apoptosis were performed in triplicate and were repeated at least three times, and the results are expressed as means±SD.
(PDF)
