# Supplementary figures and images for: LRP16 Integrates into NF-κB Transcriptional Complex and Is Required for Its Functional Activation
Source: PLoS One. 2011 Mar 31;6(3):e18157. doi: 10.1371/journal.pone.0018157 (PMC3069058; doi:10.1371/journal.pone.0018157)

**Figure S1**

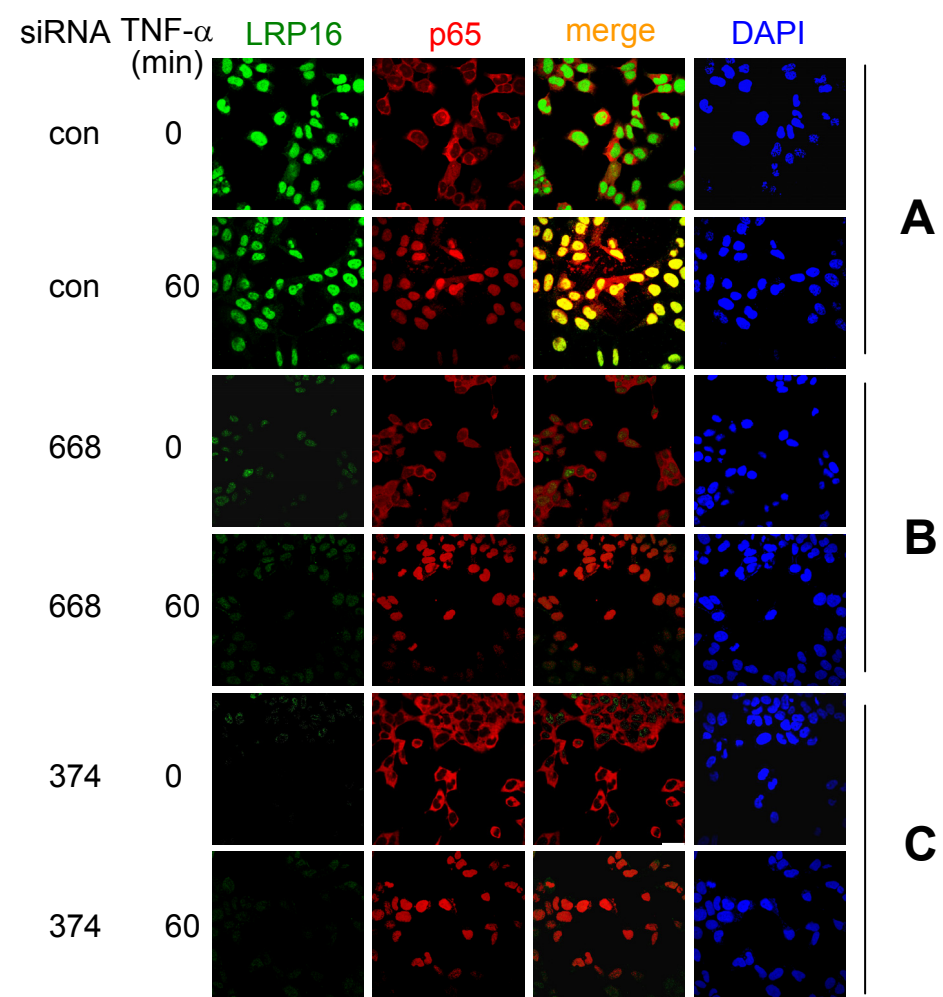

Supplement: Figure S1 — LRP16 and NF-κB p65 colocalizes in the nuclei of 293T cells in response to TNF-α stimulation. (A–C) 293T cells were transfected with the indicated siRNAs. Forty-eight hours after transfection, cells were stimulated with 10 ng/ml TNF-α for 1 h, and stained with anti-LRP16 and anti-p65 primary antibodies followed by FITC-conjugated goat anti-mouse IgG and Alexa-Fluor 594 goat anti-rabbit IgG. The nucleus was counterstained with DAPI. Bar = 20 µm. (PDF) [file pone.0018157.s001.pdf]

**Figure S2**

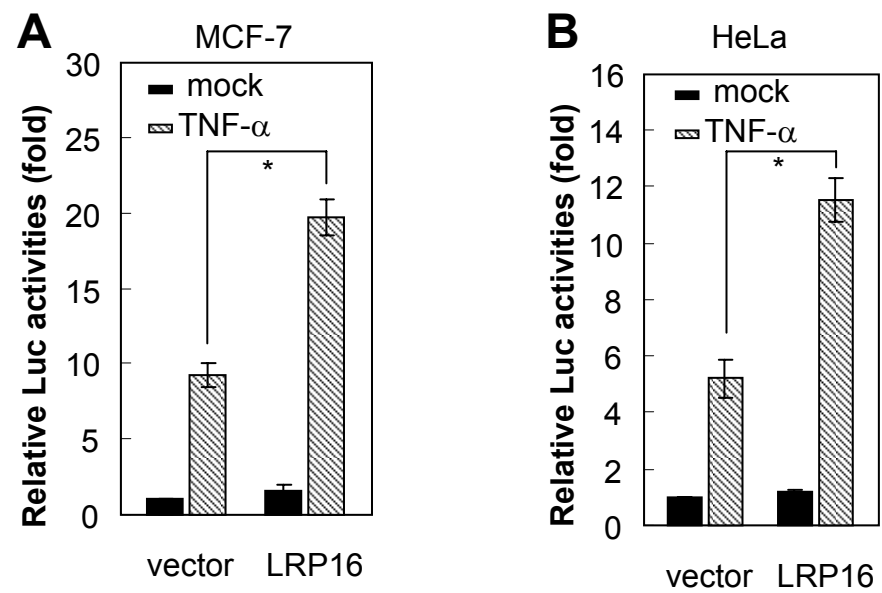

Supplement: Figure S2 — Ectopic expression of LRP16 markedly upregulates TNF-α -induced κB-luc activity. (A, B) MCF-7 and HeLa cells were cotransfected with 3×κB-luc and the indicated vectors. Forty-two hours after transfection, cells were simulated with 10 ng/ml TNF-α for 7 h before luciferase assays were performed. The relative levels of luciferase activity were normalized to the activity obtained after cotransfection of 3×κB-luc and the empty expression vector, which was arbitrarily assigned a value of 1. All experiments were performed in triplicate and were repeated at least three times, and the results are expressed as means±SD. *P<0.05. (PDF) [file pone.0018157.s002.pdf]

**Figure S3**

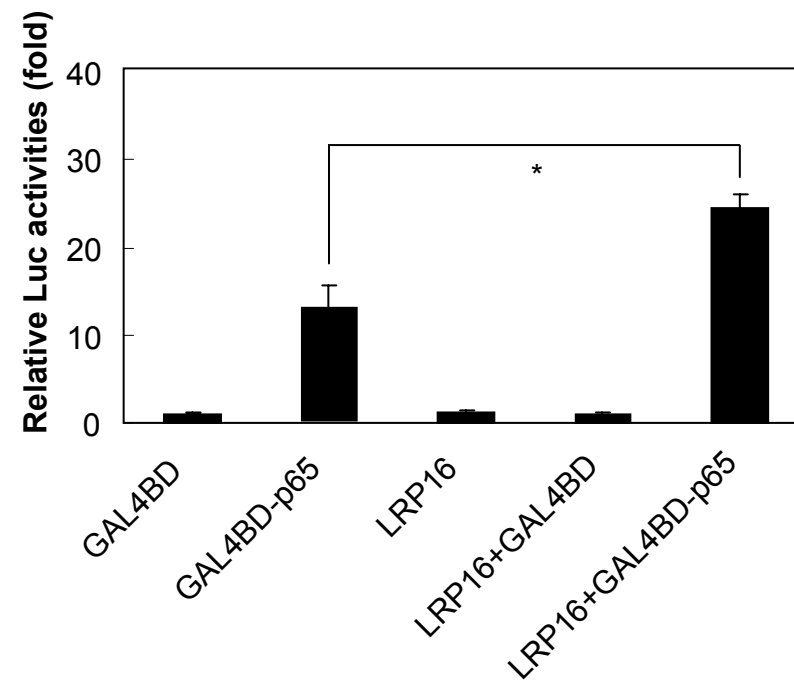

Supplement: Figure S3 — Ectopic expression of LRP16 markedly upregulates the GAL4BD-p65 induction of GAL4-luc activity. Gal4-luc was cotransfected into 293T cells with the indicated vectors. pRL-SV40 was used as transfection control. Forty-two hours after transfection, cells were lysed for luciferase assays. The relative levels of luciferase activity were normalized to the activity obtained after cotransfection of GAL4-luc and the empty expression vector, which was arbitrarily assigned a value of 1. Data represent means ± SD (error bars) of at least three independent experiments. *P<0.05. (PDF) [file pone.0018157.s003.pdf]

Figure S4

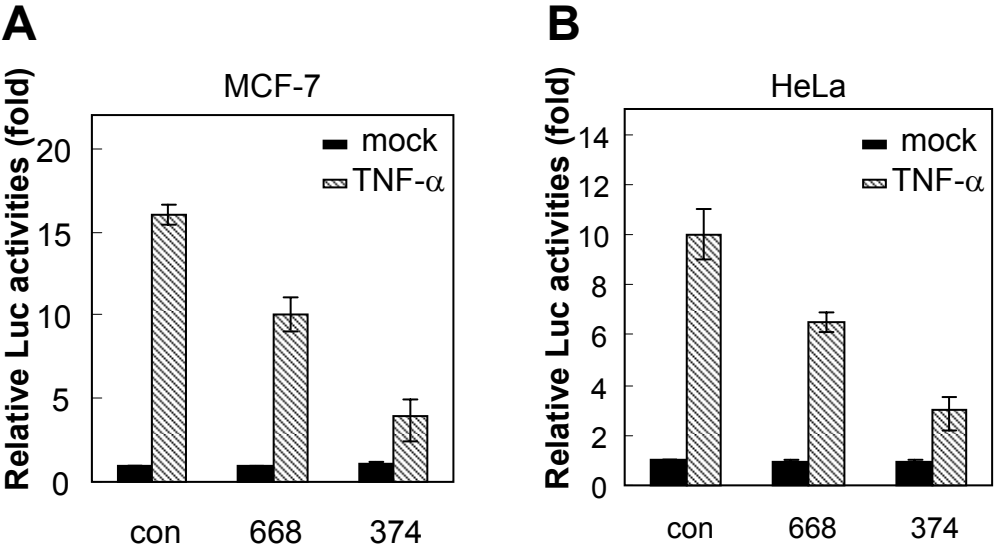

Supplement: Figure S4 — Knockdown of LRP16 markedly attenuates NF-κB transcriptional activity. (A, B) MCF-7 and HeLa cells were cotransfected with 3×κB-luc, siRNA and vectors as indicated. Forty-two hours after transfection, cells were stimulated with 10 ng/ml TNF-α for 7 h and then luciferase assays were performed. The relative levels of luciferase activity were normalized to the activity obtained after cotransfection of 3×κB-luc and the empty expression vector, which was arbitrarily assigned a value of 1. All experiments were performed in triplicate and were repeated at least three times, and the results are expressed as means±SD. (PDF) [file pone.0018157.s004.pdf]

Figure S5

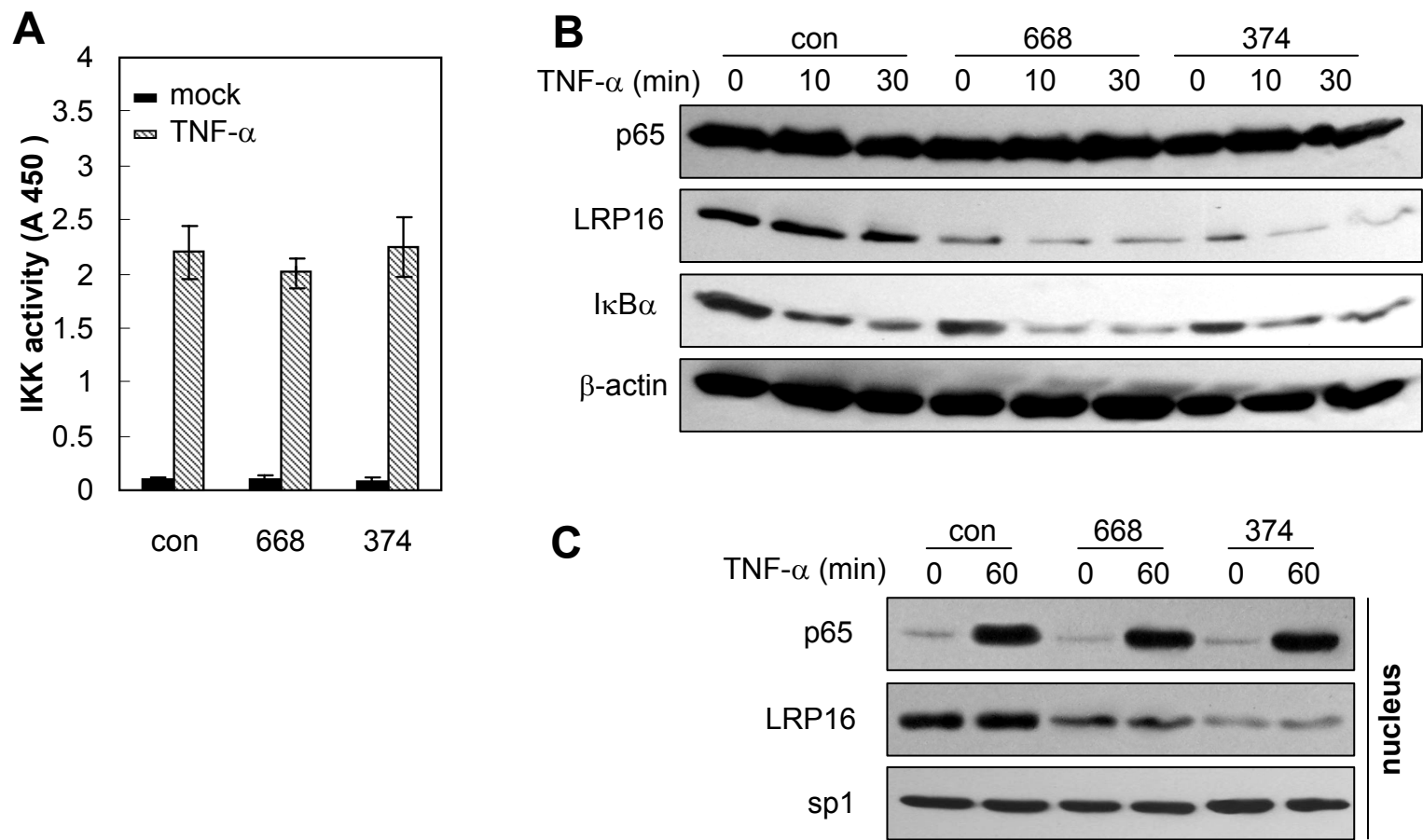

Supplement: Figure S5 — Knockdown of endogenous LRP16 expression in 293T cells does not affect TNF-α-stimulated IKK activity and nuclear translocation of NF-κB. (A) 293T cells were transfected with the indicated siRNAs. Forty-eight hours after transfection, cells were treated with 10 ng/ml TNF-α for 15 min, and lysed for IKK activity analysis. (B) 293T cells were transfected with the indicated siRNAs, treated with TNF-α for the indicated time points, lysed and the lysates were subjected to immunoblotting with the indicated antibodies. (C) 293T cells were transfected with the indicated siRNAs, treated with TNF-α for the indicated time points, nuclear extracts were used for immunoblotting analysis with the indicated antibodies. (PDF) [file pone.0018157.s005.pdf]

**Figure S6**

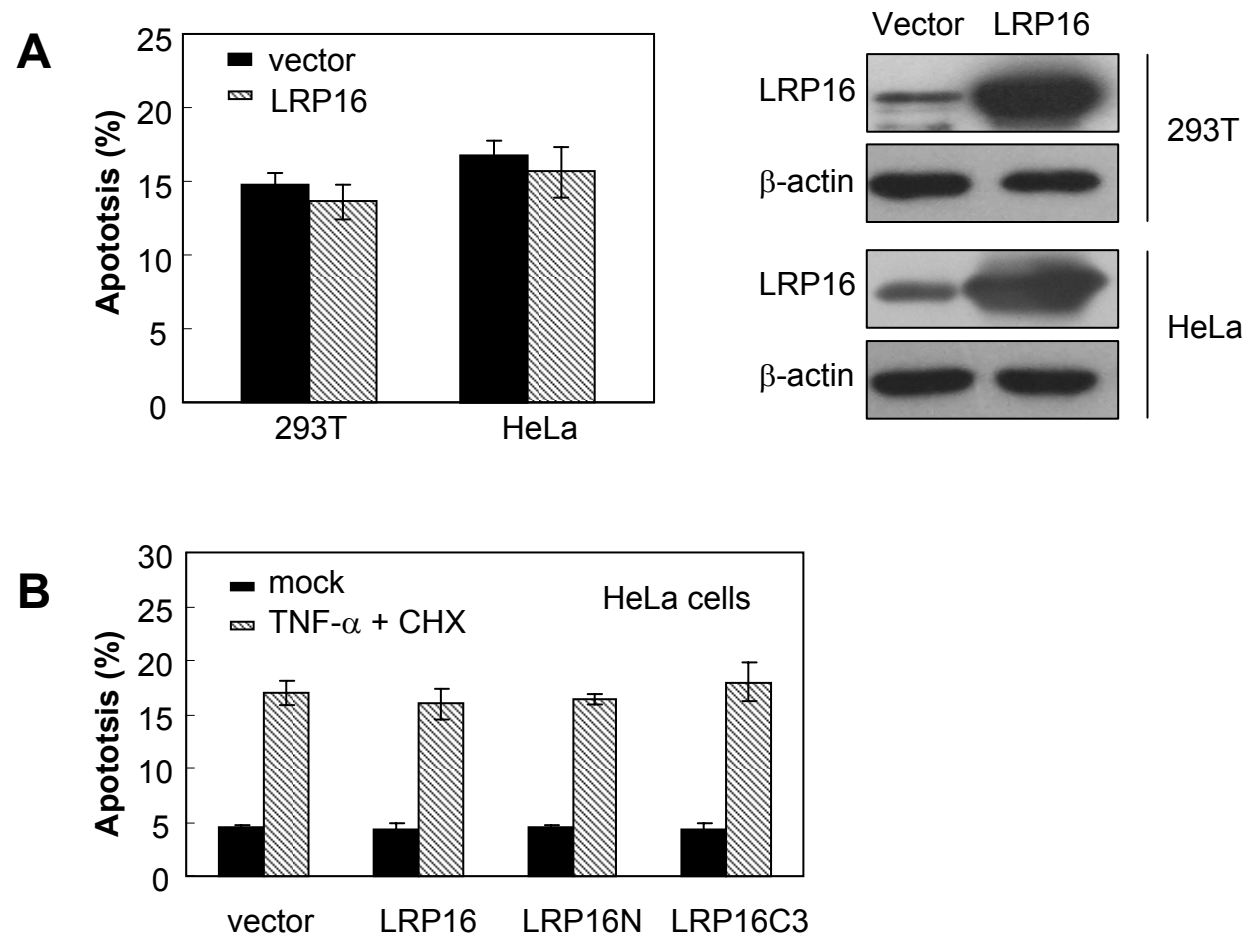

Supplement: Figure S6 — Ectopic expression of LRP16 or LRP16 mutants do not significantly promote TNF-α-induced cell apoptosis. (A, B) 293T and HeLa cells that had been transfected stably with LRP16 or the indicated LRP16 mutants were treated with TNF-α plus CHX or left untreated for 22 h. Annexin V assays were performed to monitor cell apoptosis. (PDF) [file pone.0018157.s006.pdf]

Figure S7

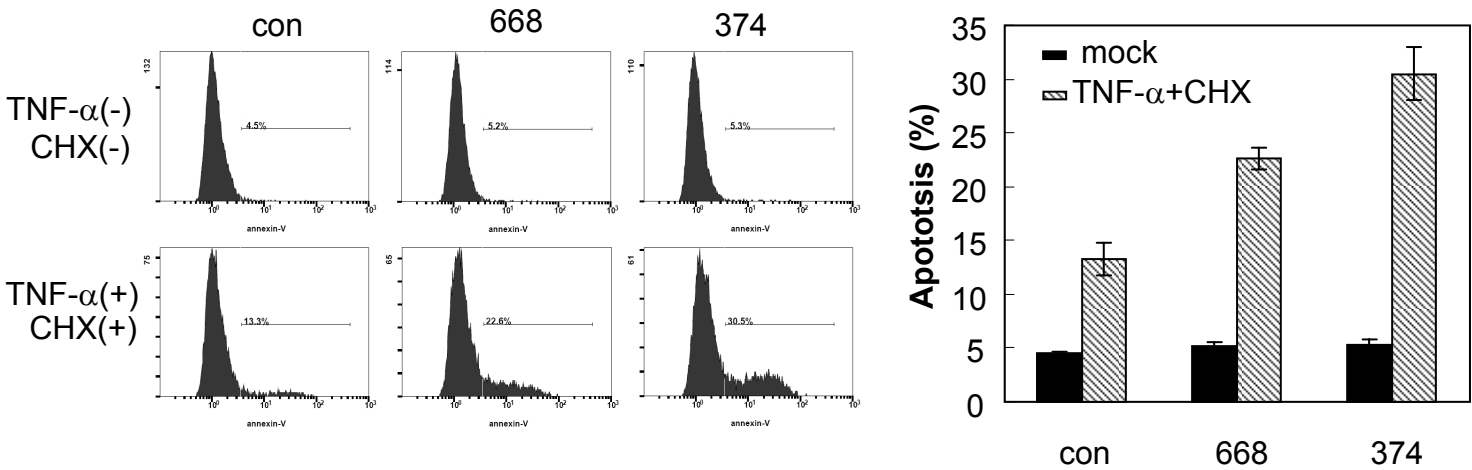

Supplement: Figure S7 — Knockdown of LRP16 sensitizes TNF-α-induced cell apoptosis. HeLa cells were transfected with the indicated siRNAs. Forty-eight hours later, cells were treated with TNF-α plus CHX or left untreated for 22 h. The percentage of apoptotic cells was monitored by annexin V staining followed by FACS analysis. Experiments to analyze apoptosis were performed in triplicate and were repeated at least three times, and the results are expressed as means±SD. (PDF) [file pone.0018157.s007.pdf]
